# Expression of overall survival-EMT-immune cell infiltration genes predict the prognosis of glioma

**DOI:** 10.1016/j.ncrna.2024.02.003

**Published:** 2024-02-16

**Authors:** Lei Zheng, Jin-jing He, Kai-xiang Zhao, Ya-fei Pan, Wei-xian Liu

**Affiliations:** a Department of Breast Surgery, Zhejiang Hospital, Hangzhou, Zhejiang Province, 310000, PR China; b Department of Operating Room, Zhejiang Hospital, Hangzhou, Zhejiang Province, 310000, PR China; c Department of Thoracic Surgery, Zhejiang Hospital, Hangzhou, Zhejiang Province, 310000, PR China; d Department of Anesthesiology, Zhejiang Cancer Hospital, Hangzhou, Zhejiang Province, 310000, PR China; e Department of Neurosurgery, Zhejiang Hospital, Hangzhou, Zhejiang Province, 310000, PR China

**Keywords:** Glioma, EMT, Tumor immune microenvironment, Prognosis, Individualized therapy

## Abstract

This study investigates the crucial role of immune- and epithelial-mesenchymal transition (EMT)-associated genes and non-coding RNAs in glioma development and diagnosis, given the challenging 5-year survival rates associated with this prevalent CNS malignant tumor. Clinical and RNA data from glioma patients were meticulously gathered from CGGA databases, and EMT-related genes were sourced from dbEMT2.0, while immune-related genes were obtained from MSigDB. Employing consensus clustering, novel molecular subgroups were identified. Subsequent analyses, including ESTIMATE, TIMER, and MCP counter, provided insights into the tumor microenvironment (TIME) and immune status. Functional studies, embracing GO, KEGG, GSVA, and GSEA analyses, unraveled the underlying mechanisms governing these molecular subgroups. Utilizing the LASSO algorithm and multivariate Cox regression, a prognostic risk model was crafted. The study unveiled two distinct molecular subgroups with significantly disparate survival outcomes. A more favorable prognosis was linked to low immune scores, high tumor purity, and an abundance of immune infiltrating cells with differential expression of non-coding RNAs, including miRNAs. Functional analyses illuminated enrichment of immune- and EMT-associated pathways in differentially expressed genes and non-coding RNAs between these subgroups. GSVA and GSEA analyses hinted at abnormal EMT status potentially contributing to glioma-associated immune disorders. The risk model, centered on OS-EMT-ICI genes, exhibited promise in accurately predicting survival in glioma. Additionally, a nomogram integrating the risk model with clinical characteristics demonstrated notable accuracy in prognostic predictions for glioma patients. In conclusion, OS-EMT-ICI gene and non-coding RNA expression emerges as a valuable indicator intricately linked to immune microenvironment dysregulation, offering a robust tool for precise prognosis prediction in glioma patients within the OBMRC framework.

## 1 Introduction

Gliomas originating from glial or precursor cells and manifesting as astrocytoma, oligodendroglioma, ependymoma, or oligoastrocytoma [1,2], constitute 81% of central nervous system (CNS) malignancies [3]. The 2016 ″World Health Organization Classification of Tumors of the Central Nervous System” classifies gliomas into four grades, with grades 1 and 2 denoting low-grade and grades 3 and 4 representing high-grade gliomas [4]. The prognosis worsens with higher glioma grades; low-grade gliomas boast a 47% 10-year survival rate and a median survival of 11.6 years [5]. Conversely, grade 3 glioma patients face a median overall survival (OS) of about 3 years, while grade 4 glioma patients experience a bleaker median OS of 15 months [6]. Despite significant strides in glioma treatment and research, gliomas' high heterogeneity and immunogenicity have hindered substantial improvements in overall survival [7]. Notably, effective clinical therapies for grade 4 gliomas remain elusive [8].

Increasingly, studies have highlighted the pivotal role of epithelial-mesenchymal transformation (EMT) as a crucial biological event in tumorigenesis and development, particularly exerting a decisive influence on tumor metastasis [[9], [10], [11], [12], [13]]. EMT represents a phenomenon where epithelial cells undergo transition, losing cell polarity and the characteristics of epithelial tissue to acquire a mesenchymal phenotype [[14], [15], [16]]. This transformative process is characterized by heightened migration capability, invasiveness, anti-apoptotic traits, and the synthesis of extracellular matrix components capable of degrading the basement membrane [[17], [18], [19]]. The progression of EMT unfolds at various stages of tumor development, emerging as a critical event in the carcinogenic process [20]. Despite its recognized significance, the specific mechanism of EMT in gliomas remains enigmatic.

While immunotherapy has demonstrated success in various cancer types, the extension of such success to management of glioma patients and their care has not been very promising [21,22]. This challenge underscores the necessity for a more nuanced understanding of the glioma microenvironment. Further investigation into the immune-related microenvironment of gliomas is essential to elucidate the limitations of existing immunotherapies in treating glioblastoma. Recent research emphasizes that the immune composition and status are specific to individual cancer lineages [23,24]. Gliomas exhibit a distinctive immune microenvironment primarily infiltrated by myeloid-derived cells, such as macrophages/microglia, expressing immunosuppressive phenotypes. These cells have been implicated in promoting tumor progression and are associated with diminished survival rates [25,26].

In this study, we used the microarray expression data of glioma patients in CGGA database (http://www.cgga.org.cn) [27,28], combined with EMT gene in dbEMT2.0 database (http://dbemt.bioinfo-minzhao. org/) [29] and immune infiltration related genes in MSigDB database (http://www.broadinstitute.org/msigdb) [[30], [31], [32]], to explore the possible biological mechanism of overall survival-EMT-immune cell infiltration related genes (OS-EMT-ICI genes) in the occurrence of glioma and its impact on the prognosis of glioma patients by R language and online biological information analysis tools (such as Metascape, etc.).

## 2 Materials and methods

### 2.1 CGGA dataset download

We obtained clinical data, encompassing RNA sequencing data and survival information of glioma patients necessary for this study, from the Chinese Glioma Genome Atlas (CCGA) website (http://www.cgga.org.cn/download.jsp). The training set, identified by dataset ID mRNAseq_325, utilizes mRNA sequencing data from the Illumina HiSeq 2000 or 2500 platform, totaling 325 samples. The validation set, associated with dataset ID mRNA array_301, employs mRNA microarray data from the Agilent Whole Human Genome (Array) platform, totaling 301 samples. For this study, our inclusion criteria was: (a) Glioma diagnosis; (b) Information on complete clinical information (which includes data on age, gender, survival etc) and gene expression and (c) In the case of paired samples, only one was included. As for the exclusion criteria, we had following considerations: (a) No glioma diagnosis; (b) Missing information on clinical characteristics; (c) Undetectable gene expression for half of the genes and (d) biased gene expression values. The training set utilizes 325 samples meeting the specified criteria, while the validation set comprises 301 samples adhering to the same criteria. Demographic and clinical characteristics for both sets are detailed in Table 1.

**Table 1:** Patient characteristics in training and the validation cohort.

Characteristics	Train (N = 325)	Validation (N = 301)	Total (N = 626)	P value
Gender				0.55
Female	122 (19.49%)	121 (19.33%)	243 (38.82%)	
Male	203 (32.43%)	180 (28.75%)	383 (61.18%)	
Age				
Mean ± SD	42.94 ± 11.95	42.42 ± 11.83	42.69 ± 11.89	
Median [min-max]	42.00 [8.00,79.00]	42.00 [12.00,70.00]	42.00 [8.00,79.00]	
PRS_type				2.9E-06
NA	4 (0.64%)	3 (0.48%)	7 (1.12%)	
Primary	229 (36.58%)	264 (42.17%)	493 (78.75%)	
Recurrent	62 (9.90%)	23 (3.67%)	85 (13.58%)	
Secondary	30 (4.79%)	11 (1.76%)	41 (6.55%)	
Grade				0.21
NA	4 (0.64%)	3 (0.48%)	7 (1.12%)	
WHO II	103 (16.45%)	117 (18.69%)	220 (35.14%)	
WHO III	79 (12.62%)	57 (9.11%)	136 (21.73%)	
WHO IV	139 (22.20%)	124 (19.81%)	263 (42.01%)	
OS				
Mean ± SD	1451.07 ± 1472.63	1659.99 ± 1604.26	1550.64 ± 1539.01	
Median [min-max]	705.00 [19.00,4809.00]	812.00 [21.00,5159.00]	759.50 [19.00,5159.00]	
Censor (alive = 0; dead = 1)				0.23
DEAD	220 (35.14%)	187 (29.87%)	407 (65.02%)	
LIVE	96 (15.34%)	100 (15.97%)	196 (31.31%)	
NA	9 (1.44%)	14 (2.24%)	23 (3.67%)	
IDH_mutation_status				0.06
Mutant	175 (27.96%)	134 (21.41%)	309 (49.36%)	
NA	1 (0.16%)	2 (0.32%)	3 (0.48%)	
Wildtype	149 (23.80%)	165 (26.36%)	314 (50.16%)	
1p19q_codeletion_status				5E-68
Codel	67 (10.70%)	16 (2.56%)	83 (13.26%)	
NA	8 (1.28%)	209 (33.39%)	217 (34.66%)	
Non-codel	250 (39.94%)	76 (12.14%)	326 (52.08%)	
MGMTp_methylation_status				0.0002
NA	19 (3.04%)	15 (2.40%)	34 (5.43%)	
methylated	157 (25.08%)	99 (15.81%)	256 (40.89%)	
un-methylated	149 (23.80%)	187 (29.87%)	336 (53.67%)	

#### 2.1.1 EMT-related gene set

The gene set relevant to EMT required for this study was obtained from dbEMT2.0 (http://dbemt.bioinfo-minzhao.org/index.html), comprising 1184 EMT-related genes [29].

#### 2.1.2 Immune-related gene set

We retrieved immune-related gene set from MSigDB (http://www.gsea-msigdb.org/gsea/msigdb/, systematic name: M1045), consisting of 908 immune-related genes [30,31].

#### 2.1.3 Acquisition of overall survival-related genes in the training set

Utilizing the survival package in R (3.6.3) and incorporating overall survival time and survival status, each gene's prognostic significance was evaluated through the univariate Cox regression method. Genes associated with the overall survival of glioma patients were identified based on a log-rank level of <0.001.

#### 2.1.4 Acquisition of OS-EMT-ICI genes in glioma patients

Following data processing, three gene sets were derived: OS genes, EMT genes, and ICI genes. The Venn diagram, an R-based mapping software package (version 3.6.3), was employed to intersect these gene sets, obtaining OS-EMT-ICI genes of glioma patients. The Venn diagram was created using the online tool jvenn (Project home page: http://bioinfo.genotoul.fr/jvenn) [33].

### 2.2 Identification of molecular subsets and TIMER [34]

The clustering of the expression matrix of the 77 OS-EMT–ICI–related genes acquired earlier was conducted using the consensus clustering package in R (3.6.3). Subsequently, the Estimation of Stromal and Immune Cells in Malignant Tumor Tissues using Expression Data (ESTIMATE) algorithm was applied to compute the stromal score, immune score, and tumor purity [35].

### 2.3 Immune analysis

To assess immune infiltration, we used Tumor Immune Estimation Resource (TIMER: https://cistrome.shinyapps.io/timer/) [34,36] to assess the abundance of immune infiltrating cell types (B cell, macrophage, dendritic cell (DC), neutrophil cell, CD4 T cell, CD8 T cell). Then, we carried out Gene set variation analysis (GSVA) employing the R package GSVA to determine the enrichment score of each sample within the gene set. The gene rank was predefined, as explained by David A Barbie method. The h.all.v7.4.symbols.gmt subset from the Molecular Signatures Database [[30], [31], [32],^37^] was then evaluated to reveal mechanistic information. Finally, we calculated the enrichment score for each sample in each gene set by setting the gene set limits as 5–5000. Resulting in the acquisition of the enrichment score matrix.

### 2.4 Functional analysis

Initially, we identified differentially expressed genes (DEGs) between clusters by employing limma package in R (version 3.6.3). Then, we conducted Gene Ontology (GO) analysis and Kyoto Encyclopedia of Genes and Genomes (KEGG), using the “cluster profiler” package in R (version 3.6.3) to determine the enrichment of relevant pathways. Visualization of these pathways was accomplished using the online software Metascape (https://metascape.org) [38]. The gene set, based on “Go: biological process (BP)" from the Molecular Signatures Database, was downloaded to perform Gene Set Variation Analysis (GSVA) using the “GSVA” package in R (version 3.6.3) to explore signal pathway changes between the two clusters. Concurrently, we used the same dataset, Gene Set Enrichment Analysis (GSEA), to assess differences between clusters.

### 2.5 Establishment and verification of risk model

**Fig. 1:**
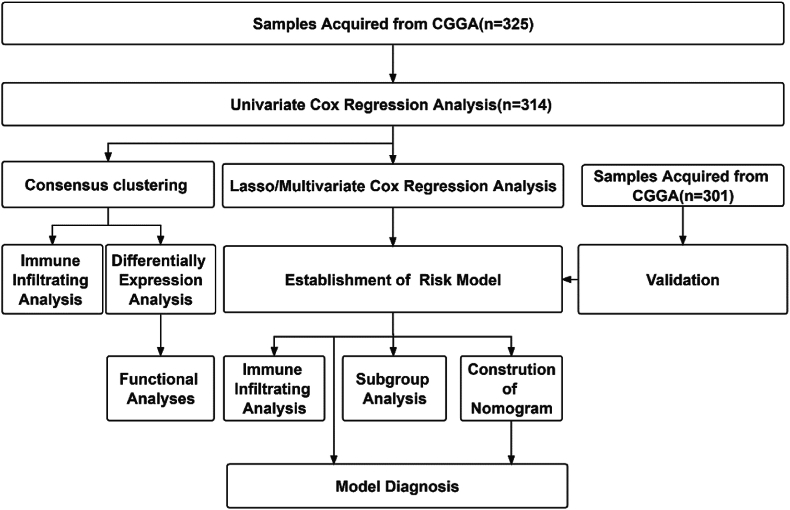
A snap shot of the data analyzing process.

Prognostic genes, initially filtered using the “glmnet” package, underwent least absolute shrinkage and selection operator (LASSO) analysis. The optimal value for the minimum lambda (λ) was determined. Multivariate Cox regression analysis was employed to select genes for constructing the risk model. The risk score calculation for each patient in both the training set and verification set followed the formula: Risk_Score = 0.1716 × expression value of MYD88 + 0.3235 × expression value of TXN +0.1258 × expression value of PIK3R3 + 0.3468 × expression value of MAPK7 + 0.3647 × expression value of MTOR +0.0049 × expression value of BCL2L1 - 0.2550 × expression value of STIM1 - 0.0695 × expression value of PSTPIP1. Subsequently, patients were categorized into high-risk and low-risk groups based on the median value. The model's prediction efficiency was assessed using ROC and martingale residual methods. The entire data analysis process is illustrated in Fig. 1.

### 2.6 Statistical analysis

R (version 3.6.3) and GraphpadPrism (version 8.0.1) were employed for statistical analysis, and TBtools [39] facilitated visualization. Survival analysis utilized the Kaplan-Meier method, while the “survivalROC” R package assessed the time-dependent receiver operating characteristics (ROC) for evaluating the prediction performance of the risk model. Subgroup analysis was also conducted using age of patients, gender, and methylation of MGMT. Statistical analysis for two data groups employed Student's t-test, while for three or more groups, one-way ANOVA analysis was selected. For our analysis, we considered p-values <0.05 to be statistically significant.

## 3 Results

### 3.1 OS-EMT–ICI–genes in glioma patients

**Fig. 2:**
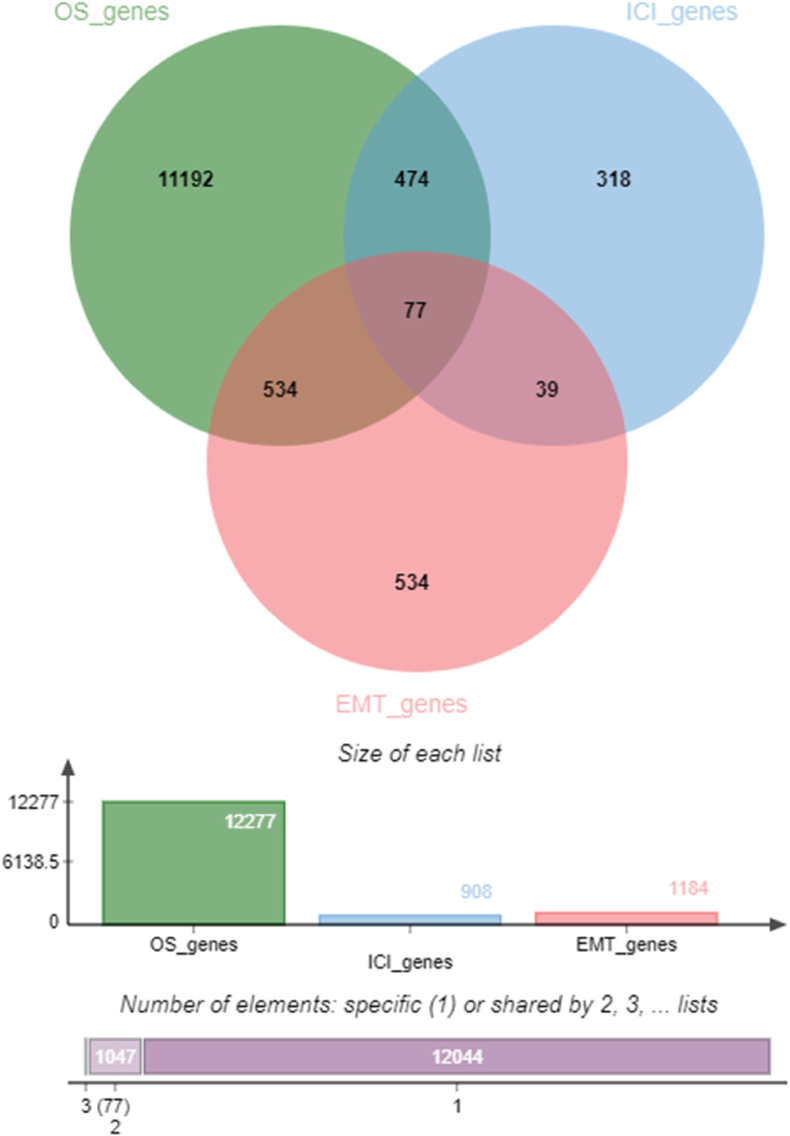
Acquisition of OS-EMT-ICI genes. OS, overall survival; EMT, epithelial mesenchymal transition; ICI, immune cell infiltration.

The training cohort dataset from the Chinese Glioma Genome Atlas (CCGA) database provided expression data for 24,326 genes and clinical information, including overall survival time and pathological details for 325 samples. Initial survival analysis, integrating overall survival (both time and status) and gene expression data for all genes through the survival package in R (3.6.3), identified 12,277 genes significantly related to glioma patient survival (log-rank <0.001). Subsequently, gene sets related to EMT from the dbEMT2.0 website and immune cell infiltration from the MSigDB website intersected with the survival-related gene set, yielding 77 genes related to overall survival-EMT-immune cell infiltration in glioma patients. Finally, Venn diagram was generated using the online tool jvenn (Fig. 2). The 77 identified genes include ADAM17, AGER, AKT1, AKT2, BCL2L1, BIRC2, BTRC, CAPZA1, CCR2, CD14, CD274, CD36, CD44, CDC42, CREB1, CRK, CRKL, CSK, CTSL, CUL3, EGR1, EIF4E, EIF4G1, ELK1, EP300, FOXO3, FOXO4, GSK3A, HGF, HSPA5, IKBKG, IL18, IL1B, IL6, L6R, IRF8, IRS2, ISG15, ITGB1, JAK3, JUN, LGALS3, LYN, MAP3K3, MAP3K7, MAPK1, MAPK14, MAPK3, MAPK7, MAPK8, MDM2, MRC2, MTOR, MYD88, PDCD1, PIK3R1, PIK3R3, PRKCQ, PSTPIP1, PTPN6, RAC1, RAF1, RELA, SHC1, SKP1, SKP2, SOCS3, SQSTM1, STAT1, STAT3, STAT5A, STIM1, TAB1, TBK1, TRIM11, TXN, UBE3C. Additionally, a distinct pattern of non-coding RNA expression was observed, with differentially expressed miRNAs (Results not shown).

### 3.2 OS-EMT–ICI–genes-based detection of individual subtypes

**Fig. 3:**
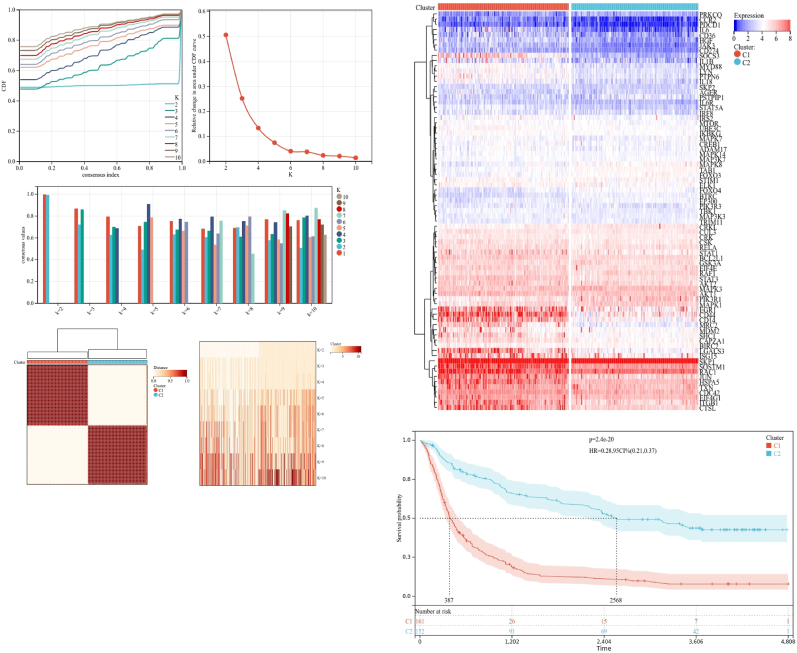
Consensus cluster. (A–C) K = 2 was identiﬁed the optimal value for consensus clustering, (D) heatmap visualizing the expression of OS-EMT-ICI gens in the two subgroups, (E) survival curve of the patients in the two subgroups.

Utilizing the consensus clustering approach, glioma patients from training cohort were stratified into subgroups based on the 77 prognostic genes derived from univariable Cox analysis. Optimal clustering stability was achieved at K = 2 (Fig. 3A–C), with 165 patients grouped into Cluster 1 and 160 into Cluster 2. Visualization of the expression levels of OS-EMT–ICI–Genes in the two subtypes was depicted in the heatmap (Fig. 3D), revealing significant expression differences between Cluster 1 and Cluster 2. Additionally, patients in Cluster 2 exhibited better overall survival compared to those in Cluster 1 (P = 2.4e-20; Fig. 3E). These findings indicated that the OS-EMT–ICI–Genes effectively classified glioma patients into two molecular subtypes with distinct overall survival outcomes.

### 3.3 TIME and immune status of patients

**Fig. 4:**
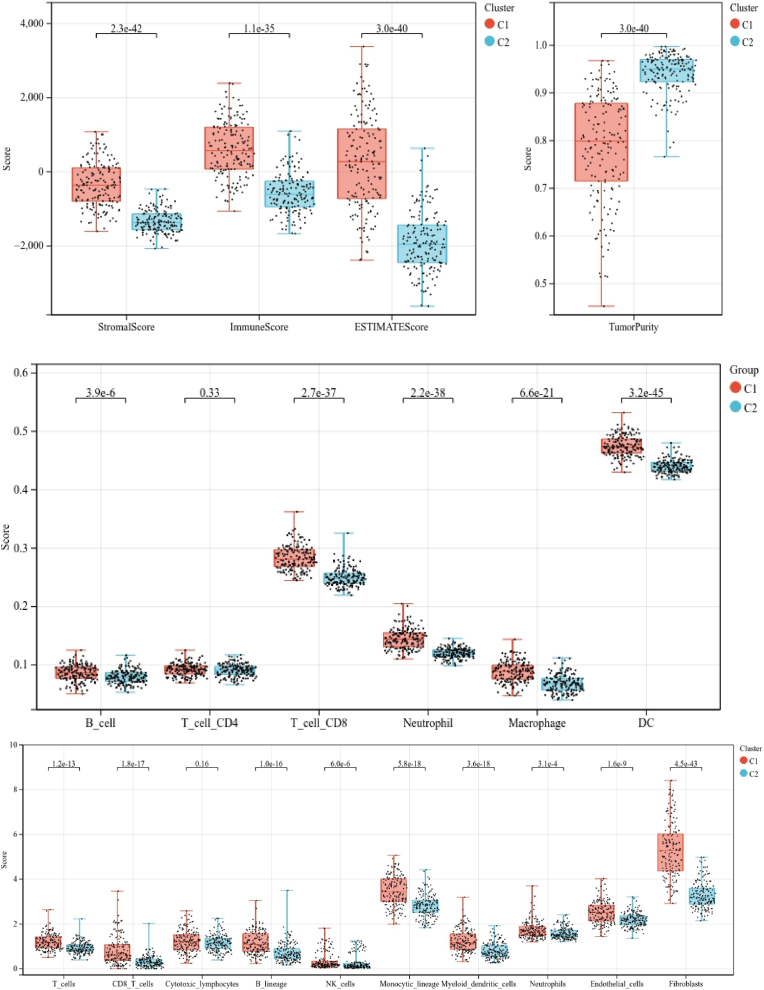
Immune analyses in the two clustered subgroups. (A) Stromal score, immune score, ESTIMATE score and (B) tumor purity calculated by ESTIMATE algorithm, (C) abundance of six immune ﬁltrating cells evaluated by TIMER, (D) immune status score obtained by MCP counter.

Immune analyses were done to find and list the differences between subtypes. Using ESTIMATE algorithm, we found that the glioma patients from cluster 1 exhibited higher stroma scores (P = 2.3e-42), immune scores (P = 1.1e-35), ESTIMATE scores (P = 3.0e-40), and lowered tumor purity (P = 3.0e-40) compared with cluster 2 (Fig. 4A and B). TIMER algorithm suggested increased B cells (P = 3.9e-6), T cell-CD8s (P = 2.7e-37), neutrophils (P = 2.2e-38), macrophages (P = 6.6e-21) and dendritic cells (DC) (P = 3.2e-45) in cluster 1 was significantly higher than in cluster 2, while no significant difference was found in the abundance of T cell-CD4 between two clusters (P = 0.33; Fig. 4C). Immune analysis done using MCP counter algorithm showed major differences between clusters (Fig. 4D). This, our analyses showed significant TIME and immune status differences between the subtypes.

### 3.4 DEGs and functional analyses

**Fig. 5:**
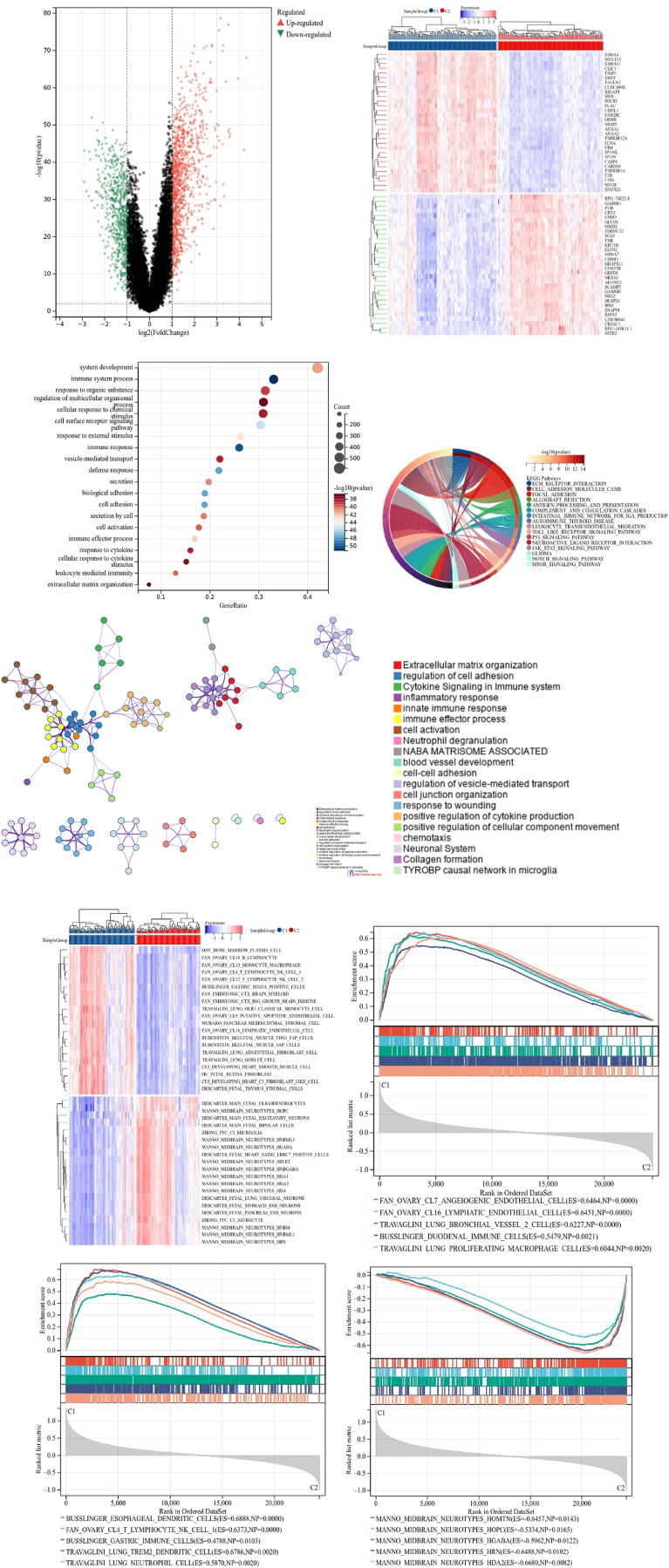
Differentially expressed genes (DEGs) analysis and functional analyses. (A) Volcano plot showing the DEGs between the two subgroups, (B) circle plot visualizing the biological processes enriched by gene ontology (GO) analysis, (C) bubble diagram showing the signaling pathways enriched by Kyoto Encyclopedia of Genes and Genomes (KEGG) analysis, (D) PPI analysis of DEGs, (E) heatmap illustrating the result of GSVA analysis, (F) GSEA plots visualizing the result of GSEA analysis.

The patients’ expression profile data was analyzed using the Limma package for the listing of DEGs between groups (FDR <0.01 and logFC = 2). We identified 1709 DEGs, with 1060 genes downregulated and 649 genes upregulated in Cluster 2, as compared to Cluster 1 (Fig. 5A). Hallmark gene set enrichment analysis revealed EMT enrichment in DEGs as well as regulation of various signaling pathways, such as interferon-gamma response, TNFα-signaling via NF-κB, KRAS signaling up, inflammatory response, IL6-JAK-STAT3 signaling and the P53 pathway (Fig. 5B). KEGG analysis also identified important biological processes that were related with the immune response, such as, immune system process, cell surface receptor signaling pathway and response to external stimulus, among others (Fig. 5C). Protein-protein interaction (PPI) analysis identified twenty sub-modules closely associated with blood vessel development and immune response, suggesting a connection between immune and EMT processes in glioma development (Fig. 5D). Further we explored relationship between enriched pathways and glioma prognosis using GSVA and GSEA analyses, revealing differential expression of biological process gene sets associated with immune processes and neurotypes between the two clusters (Fig. 5E). GSEA analysis demonstrated that hallmark gene sets related to endothelial-mesenchymal transition, angiogenesis, IL6-STAT3-signaling, and G2M checkpoint were highly expressed in Cluster 1, while neurotype cell-related gene sets were expressed predominantly in Cluster 2 (Fig. 5F and G). The two clusters also have differentially expressed non-coding RNAs, particularly miRNAs (Results not shown), and there expression needs to be experimentally verified in further studies. These findings highlight the correlation between the expression of OS-EMT ICI-genes and dysregulation of immune and EMT processes, potentially contributing to the poor prognosis of glioma patients.

### 3.5 Development of risk model based

**Fig. 6:**
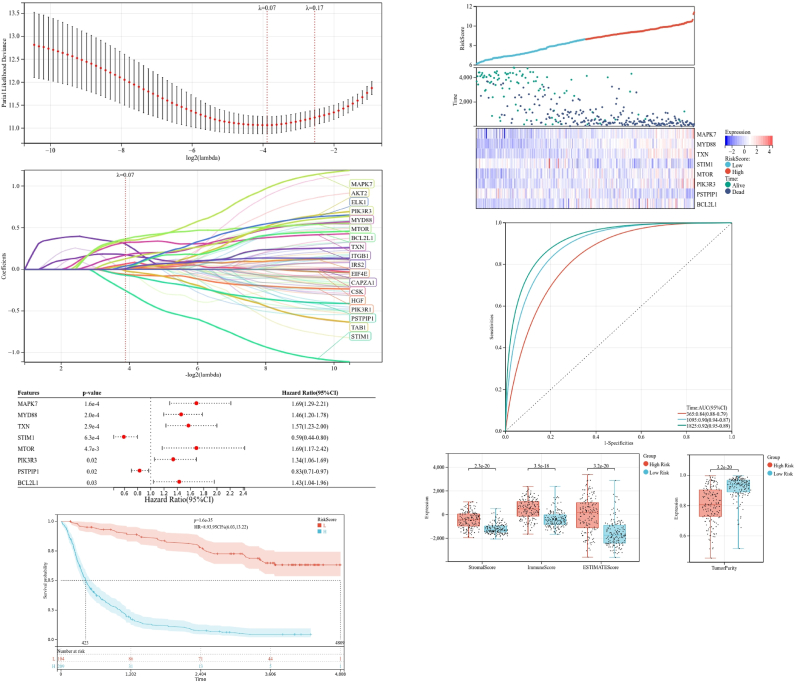
Construction of risk model in the training cohort. (A,B) LASSO analysis with minimal lambda, (C) Regression results testing the effects of eight genes to predict OS on the respective outcome, (D) survival curve of the glioma patients in the two groups, (E) distribution of survival status and risk score of glioma patients in the high and low risk groups, heatmap illustrating the expression of the eight candidate genes in the two groups, (E) time-dependent ROC curve of the risk model, (G–H) stomal score, immune score, ESTIMATE score and tumor purity calculated by ESTIMATE algorithm.

We developed a risk signature model to evaluate the prognostic predictive value of OS-EMT-ICI genes in glioma. LASSO analysis was utilized to screen potential genes for establishing the risk model, resulting in 18 genes filtered with optimal lambda values (Fig. 6A and B). From these genes, multivariate Cox analysis identified eight genes (MAPK7, MYD88, TXN, STIM1, MTOR, PIK3R3, PSTPIP1, and BCL2L1) to construct the risk model. STIM1 and PSTPIP1 were protective genes, while MAKP7, MYD88, TXN, MTOR, PIK3R3, and BCL2L1 were risk genes (Fig. 6C). Kaplan-Meier analysis revealed these genes to be independent prognostic markers for glioma (Fig. 6D). We were able to classify patients into patients with high-risk vs. patients with low-risk (Fig. 6E). High risk patients tended to have relatively higher expression of six candidate genes, with lowered expression of two protective genes. With such differences, low-risk patients exhibited an overall better survival among the two groups (Fig. 6E).

Further, the time-dependent ROC curve (Fig. 6F) indicated an acceptable assessment result. Time-dependent This analysis also revealed the precise predictive capacity for 5 years for constructed risk model, with the AUC of the ROC curve for 1, 3, and 5 years being 0.84, 0.90, and 0.92, respectively (Fig. 6F). We used ESTIMATE algorithm for the evaluation of TIME between groups. The results showed that low-risk patients had comparatively lower stromal score (P = 2.3e-20), immune score (P = 3.5e-18) and ESTIMATE score (P = 3.2e-20) (Fig. 6G), however, the tumor purity in this low-risk group was significantly elevated (Fig. 6H). Our analysis thus outlined the benefits of risk model in terms of patients prognosis and the tumor immune microenvironment.

### 3.6 Constructed risk model independence

**Fig. 7:**
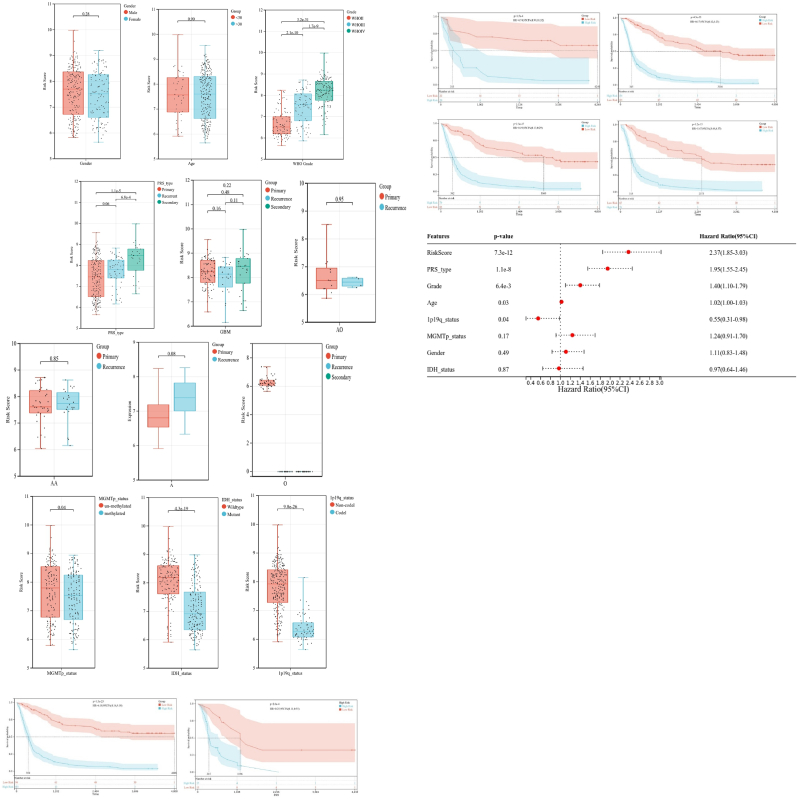
Association of risk score and clinical characteristics (A–D). No signiﬁcant difference was identiﬁed in patients with different age (A), sex (B), MGMT status (C) and between primary and recurrent glioma(D). Independence analysis of the risk model (E–J). Survival curve of patients regrouped according to age (E,F), sex (G,H), and metastasis (I,J). The risk score of constructed risk model was an independent predictive marker for glioma patients(K).

Moreover, we investigated the relationship between clinical features and risk score and thus assessed constructed risk model's independence, using subgroup and regression analysis. We did not observe any difference that was statistically significant in risk scores between patients of different genders (Fig. 7A), ages (Fig. 7B), MGMT methylated status (Fig. 7C), and recurrence within the same pathological tissue type (Fig. 7D). Additionally, when patients were grouped by age (Fig. 7E and F), gender (Fig. 7G and H) and MGMT methylated status (Fig. 7I–J), a potent predictive performance of the risk model was evident. The multivariate Cox regression analyses further established the predictive ability of constructed risk model (Fig. 7K).

### 3.7 Results from the verification cohort

**Fig. 8:**
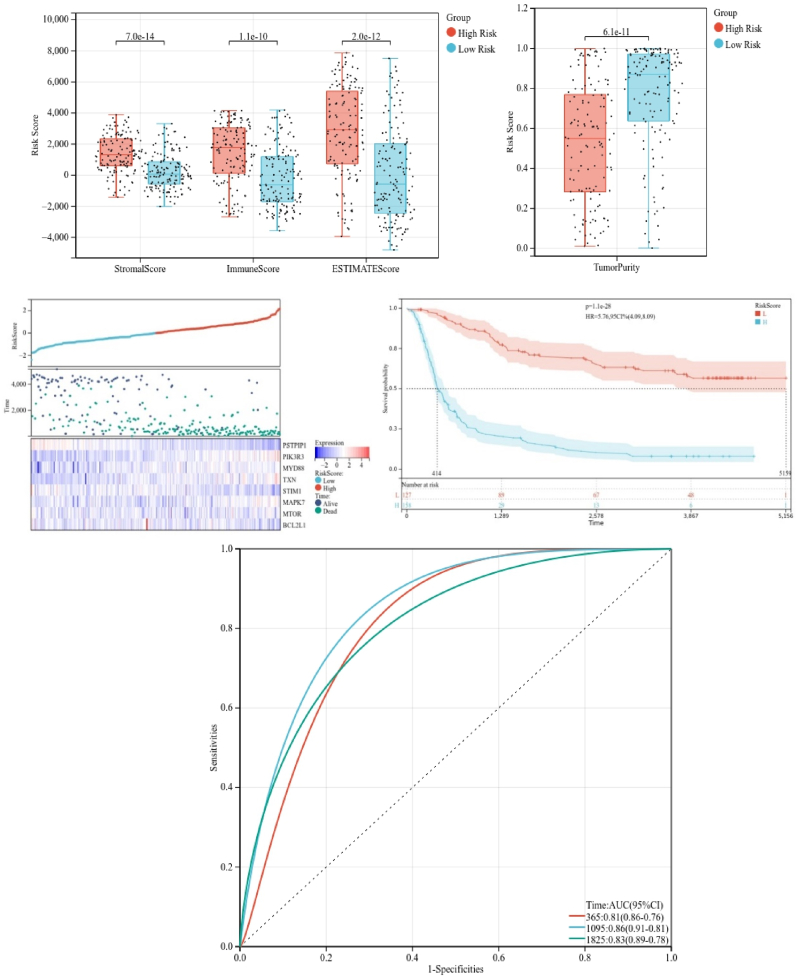
Validation of the constructed risk model in the veriﬁcation cohort. (A) Distribution of survival status and risk score, heatmap illustrating the expression of three candidate genes in the veriﬁcation cohort, (B) survival curve of the patients in the high and low risk groups in the veriﬁcation cohort, (C) ROC curve of the risk model in the veriﬁcation cohort, (D) stromal score, immune score, ESTIMATE score and tumor purity calculated by ESTIMATE algorithm.

Subsequently, we analyzed verification cohort to test the findings from our training cohort. Employing the previously outlined criteria, glioma patients were categorized into different risk groups in verification cohort (depicted in Fig. 8A), and heatmap revealed eight putative genes (Fig. 8A). We found through survival analysis that the high-risk patients had poor prognosis (P = 1.1e-28; Fig. 8B). Through ROC analysis we could confirm that risk model had optimal prediction accuracy for 1-, 3-, and 5-year survival rates (Fig. 8C). In alignment with training cohort, the low-risk group demonstrated relatively lower stromal score (P = 7.0e-14), immune score (P = 1.1e-10), and ESTIMATE score (P = 2.0e-12) (Fig. 8D), with notably higher tumor purity (P = 6.1e-11; Fig. 8D). These findings emphasized the usefulness of findings from training cohort that could be confirmed in the verification cohort.

### 3.8 Development of integrated monogram

**Figure 9:**
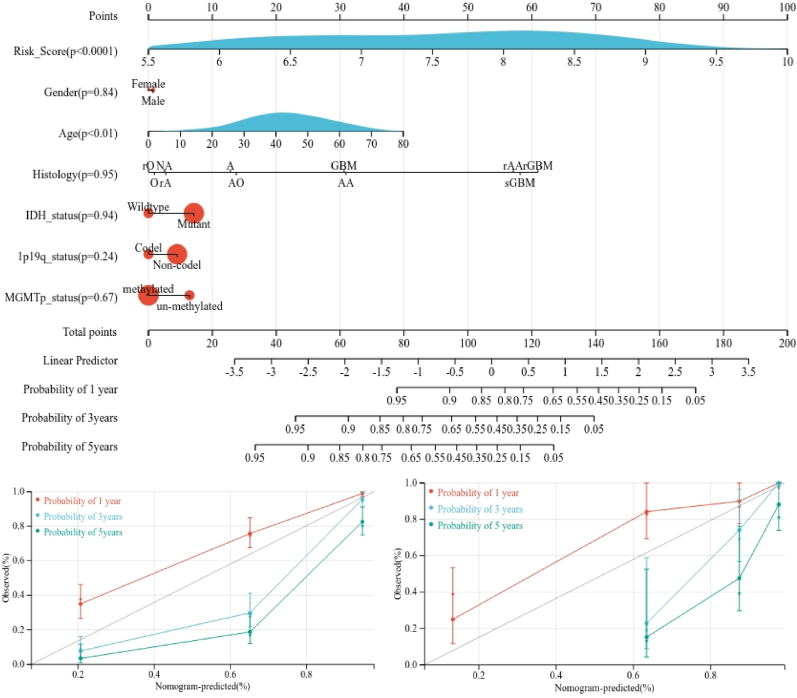
Construction and calibration of nomogram. (A) nomogram integrating risk score and clinical features, (B) calibration of the nomogram at 1, 3 and 5 years in the training cohort, (C) calibration of the nomogram at 1, 3 and 5 years in the veriﬁcation cohort.

Finally, we developed a nomogram that integrates clinical features and risk model to enhance the precision of predicting glioma patients' prognosis (depicted in Fig. 9A). The nomogram assigned specific scores to risk score and age based on their contributions to glioma prognosis. Subsequently, we validated the nomogram in both the training and verification cohorts. Regarding the model diagnosis of the nomogram, the C-index and calibration curve (Fig. 9B and C) indicated acceptable accuracy. The C-index in training cohort reached 0.8244 (95% CI: 0.7995–0.8494) and the survival closely matched the actual survival at 1, 3, and 5 years (Fig. 9B), with comparable observations in verification cohort (C index: 0.8244, 95% CI: 0.7933–0.8911, Fig. 9C). Thus, integrated nomogram accurately predicted glioma patients prognosis.

Overall, these results suggest that immune dysfunction and EMT may play pivotal roles, leading to poor glioma prognosis. The risk model, grounded in EMT and immune function, proves to be a reliable and accurate tool for prognostic prediction in glioma patients.

## 4 Discussion

Glioma, arising from specialized non-neural and glial cells has currently unfavorable prognosis for patients [40]. Existing treatments, including, surgery, chemotherapy and radiotherapy, fall short in curbing the disease's progression [41]. Despite numerous advancements in cancer therapies, only a few drugs have gained FDA approval for glioma treatment [42]. The formidable challenge in glioma treatment is the presence of blood-brain barrier that impedes penetration of most anti-tumor therapies [43,44]. Here, we identified two molecular subtypes with distinct immune and EMT characteristics. Immune analyses revealed significant abnormalities in immune functions among patients with poor prognoses, exhibiting higher stromal cell scores, immune scores, and ESTIMATE scores, along with lower tumor purity compared to patients with better prognoses.

Subsequent functional analyses unveiled the involvement of biological processes like immune response, inflammatory response, extracellular matrix, and EMT in the onset and progression of glioma. Furthermore, we established a prognostic risk model based on EMT and immune-related genes, demonstrating precise prognostic predictions for glioma patients. These findings have the potential to advance targeted therapy development for glioma and aid clinicians in making more informed treatment decisions.

Utilizing consensus clustering as a reliable method for sample classification based on gene expression matrices, we identified two molecular subgroups in glioma patients with distinct overall survival rates. Subsequent immune and functional analyses aimed to elucidate the roles of immunity and EMT in glioma. As highlighted earlier, the Tumor Immune MicroEnvironment (TIME) significantly impacts patient prognosis, with alterations in the surrounding stroma influencing tumor progression, particularly involving immune cell components [45]. Furthermore, clinical observations have linked EMT with immunosuppression in various cancer types, emphasizing the importance of understanding this interplay in the tumor microenvironment [46]. Also, EMT-TFs activation contributes to enrichment of immunosuppressive cells [47]. Leveraging the ESTIMATE algorithm, which infers tumor purity and immune and stromal cell proportions based on gene expression values, immune scores quantitatively reflect the immune components, providing insights into the TIME [35]. Tumor purity, which refers to the relative abundance of tumor cells in tumor tissue, affects prognosis [35,48]. Applying ESTIMATE to assess the TIME in the two subgroups, our results indicated that patients with better prognoses exhibited lower immune scores and higher purity, aligning with prior research findings [42,[49], [50], [51]].

Additionally, we employed TIMER and MCP counters for the evaluation of immune status. The web tool TIMER helped quantify infiltrating immune subsets [34]. The analysis from TIMER revealed that 5 of 6 immune cells had higher abundance in cluster 1, aligning with ESTIMATE results. This suggested that the activation of EMT-TFs in cluster 1 might lead to enriched immunosuppressive cells. MCP counter analysis, focusing on 11 immune-related cells, supported this notion by indicating a relatively immunosuppressive status in patients from cluster 1. These findings collectively suggest that higher immune scores, indicative of immunosuppression and the subsequent accumulation of immune cells, could be associated with unfavorable prognoses.

The synergistic findings from GO, KEGG, and PPI analyses collectively suggested the involvement of immune disorders and EMT in the onset and progression of glioma. Nonetheless, the intricate relationship between immune dysfunction and EMT status remained unclear. Further, GSVA function analysis computed signal pathways utilizing gene levels [52]. Meanwhile, GSEA analysis directly illuminated the expression tendencies of gene sets in subgroups [37]. In this study, GSEA results indicated the accumulation of immune cell inhibition and abnormal EMT status in cluster 1. These findings suggest that EMT may play a role in immune regulation in glioma and could be associated with the poor prognosis of patients.

Summing up the aforementioned findings, it is reasonable to infer that immune dysfunction and an abnormal EMT state contribute to the disruption of TIME, leading to a poor prognosis in glioma. As highlighted earlier, EMT and immune-related genes are emerging markers in malignant tumors. Targeted immunotherapy strategies grounded in EMT present a promising avenue for anti-tumor treatment [16].

To further validate the impact of immune imbalance and EMT status on TIME in glioma and investigate the prognostic significance of OS-EMT-ICI genes in glioma patients, we developed a predictive model based on OS-EMT-ICI genes and verified it using validation cohort. Eight genes selected for constructing this risk model have previously been associated with development and progression of tumors. Survival analysis demonstrated that in both training and validation cohorts, the risk model predicted patients survival. Poor survival was consistently associated with elevated stromal/immune score reduced lower tumor purity. Furthermore, independence analysis and subgroup analysis indicated that OS-EMT-ICI genes-associated risk model could independently predict glioma prognosis, irrespective of age, gender, and MGMT methylation status. Finally, we established a nomogram that integrated risk score and clinical features, for the prediction of survival. All these findings affirm the prognostic predictive role of OS-EMT-ICI genes in glioma and the correlation between abnormal EMT status and TIME disorder.

In recent decades, the field of tumor immune-targeted therapy has experienced significant growth [[53], [54], [55]]. However, the efficacy of glioma treatment, particularly for glioblastoma, remains unsatisfactory [56], thus it is important to classify patients to implement personalized and targeted therapy. Studies such as ours are a viable approach to accomplish this. While previous studies have constructed risk models for glioma based on the tumor microenvironment and immune cell infiltration [57], our study offers unique advantages. Firstly, our work focused on the survival time and immune status of glioma patients, integrating the EMT status of tumor cells. Secondly, we provided insights into the underlying mechanisms. Thirdly, we clarified the impact of immune dysfunction and EMT status on the onset time and prognosis. Lastly, our study integrated another glioma dataset from CGGA as a validation cohort to validate the model. This work serves as a possible basis for future in-detail studies on glioma.

Of the two clusters identified in our study, cluster 1 patients had poor prognosis and exhibited TIME disorder, characterized by a high immune score and low tumor purity, highlighting the association of EMT and immune dysfunction with the unfavorable prognosis of glioma. The risk model based on OS-EMT-ICI genes demonstrated precise prognostic predictions for glioma. These findings underscore the correlation of EMT and immune dysfunction with TIME and emphasize their significance in future glioma therapies.

The preliminary findings from our study also suggest a role of non-coding RNAs, particularly miRNAs, in overall survival and immune status of glioma patients and this can have big implications in future clustering and treatment of glioma patients. Recently non-coding RNAs, both miRNAs [58] and long non-coding RNAs [59] have been proposed as promising RNAs in glioma. Despite the promises, there are some shortcomings in our work as well. Firstly, the need for complete molecular pathological data for glioma patients impedes our ability to establish the specific relationship and interaction between OS-EMT-ICI genes and other pathological molecules in the onset and progression of glioma. Secondly, the results were derived from bioinformatics analysis and did not demonstrate experimental validation. Based on our findings, the significance of OS-EMT-ICI genes in glioma needs further evaluation.

## 5 Conclusions

In this study, we identified two distinct glioma subtypes that differed in OS-EMT-ICI genes. Immune and functional analyses unveiled that EMT dysregulation contributes to immune suppression and cell accumulation, leading to an unfavorable prognosis. These findings can impact future studies on glioma, particularly with respect to personalized therapy.

## Data availability

The mRNA expression matrix and clinical data of glioma patients were downloaded from the CGGA database (http://www.cgga.org.cn). Immune-related gene sets were downloaded from the MSigDB database (http://www. broadinstitute.org/msigdb). The EMT-related gene sets were downloaded from the dbEMT2.0 database (http://dbemt.bioinfo-minzhao.org/). These databases are public. Details of these data are provided in the Materials Methods section.

## Conflicts of interest

The authors made no disclosures.

## Funding statement

No specific funding was disclosed.

## CRediT authorship contribution statement

**Lei Zheng:** Writing – review & editing, Writing – original draft, Validation, Methodology, Formal analysis. **Jin-jing He:** Writing – review & editing, Writing – original draft, Formal analysis, Data curation. **Kai-xiang Zhao:** Writing – review & editing, Methodology, Investigation, Formal analysis. **Ya-fei Pan:** Writing – review & editing, Formal analysis, Data curation. **Wei-xian Liu:** Writing – review & editing, Writing – original draft, Supervision, Resources, Project administration, Investigation, Conceptualization.

## Declaration of competing interest

All the authors declare that they have no conflicts of interest.
